# Positron Emission Tomography (PET) utilizing Pittsburgh compound B (PIB) detects amyloid heart deposits in hereditary transthyretin amyloidosis (ATTR).

**DOI:** 10.1186/1750-1172-10-S1-O15

**Published:** 2015-11-02

**Authors:** Björn Pilebro, Per Lindqvist, Sandra Gustafsson, Per Westermark, Torbjörn Sundström, Gunnar Antoni, Ole Suhr, Jens Sörensen

**Affiliations:** 1 Umeå University, Dep of Public Health and Clinical Medicine, 90185, Umeå, Sweden; 2 Umeå University, Dep of surgical and preoperative Sciences, 901 85, Umeå, Sweden; 3 Uppsala University, Dep of Immunology, Genetics and PAtihology, 75185, Uppsala, Sweden; 4 Umeå University, Dep of Radiation Sciences, 90185, Umeå, Sweden; 5 Uppsala University, Deep of Radiology, Oncology and Radiation Sciences, 75185, Uppsala, Sweden

## Background

Severe cardiac involvement in hereditary V30M transthyretin (TTR) amyloidosis (ATTRV30M) has been linked to amyloid containing fragmented TTR, fibril type A and “early onset” mainly neurological disease to fibrils containing only full length TTR, type B. A number of different modalities to diagnose for cardiac imaging amyloid deposition in the heart have been developed, but so far, none have been proven to specifically identify cardiac amyloid deposits in ATTR. Positron emission tomography (PET) using the tracer Pittsburgh compound B (11C-PIB) has been shown to identify cardiac amyloidosis in a small pilot. The aim of this study was to evaluate the accuracy of PIB-PET identify cardiac amyloidosis in all patients with ATTR.

## Method

Ten patients with ATTRV30M, five with each type of amyloid fibril composition, selected on criteria of having no or mild cardiac involvement, underwent 11C-PIB PET. The results were compared to results from 99Tc-DPD scintigraphy and echocardiography.

## Results

All patients had pathological 11C-PIB uptake but the pattern of uptake differed between the two groups. Patients with type B fibrils had significantly higher 11C-PIB retentions index (RI) than those with type A fibrils. Inversely, all patients with type A and none with type B fibrils had pathological 99Tc-DPD uptake. No significant differences in echocardiographic measurements were observed.

## Conclusion

11C-PIB PET identifies presence of ATTR amyloid in all patients and strengthens the argument that the fragmentation of TTR is central in the pathogenesis of ATTR cardiomyopathy. The higher 11C-PIB RI seen in patients with type B fibrils could be explained by better microcirculation or amyloid deposition pattern in the heart tissue. This phenomenon severely limits the usefulness of the method for quantifying amyloid burden.

